# The Genetic Determinants of *Listeria monocytogenes* Resistance to Bacteriocins Produced by Lactic Acid Bacteria

**DOI:** 10.3390/genes16010050

**Published:** 2025-01-03

**Authors:** Anna Zawiasa, Agnieszka Olejnik-Schmidt

**Affiliations:** Department of Food Biotechnology and Microbiology, Poznan University of Life Sciences, Wojska Polskiego 48, 60-627 Poznan, Poland; anna.zawiasa@up.poznan.pl

**Keywords:** *Listeria monocytogenes*, bacteriocins, lactic acid bacteria, resistance, genetic determinants

## Abstract

Background: *Listeria monocytogenes* is a Gram-positive bacterium responsible for listeriosis, a serious foodborne disease that can lead to serious health complications. Pregnant women, newborns, the elderly, and patients with weakened immune systems are particularly susceptible to infection. Due to the ability of *L. monocytogenes* to survive in extreme environmental conditions, such as low temperatures, high salinity, and acidity, this bacterium poses a serious threat to food production plants and is particularly difficult to eliminate from these plants. One of the promising solutions to reduce the presence of this bacterium in food products is bacteriocins as natural control agents. These are substances with antibacterial activity produced by other bacteria, mainly lactic acid bacteria (LAB), which can effectively inhibit the development of pathogens such as *L. monocytogenes*. The use of bacteriocins in the food industry is beneficial due to their natural origin, specificity of action, and consumer safety. However, the problem of resistance to these substances exists. Results: This review focuses on the mechanisms of bacteriocin resistance, such as modifications of bacteriocin docking receptors, changes in the structure of the cell wall and membrane, and the occurrence of cross-resistance to different bacteriocins. Genetic factors determining these mechanisms and strategies to cope with the problem of resistance are also presented. Conclusions: Research on this issue is crucial for developing effective preventive methods that will enable the safe and long-term use of bacteriocins in food production.

## 1. Introduction

The genus *Listeria* is an important group of microorganisms in the food industry, now consisting of 27 officially recognized species. Based on recent findings, the genus has been divided into two clades: *Listeria* sensu stricto, which includes *L. monocytogenes* and species closely related to it, and *Listeria* sensu lato, which encompasses species that are more distantly related to *L. monocytogenes* [1]. *L. monocytogenes* is pathogenic in both humans and animals [2]. On rare occasions, other *Listeria* species, such as *L. ivanovii*, *L. seeligeri,* and *L. innocua*, have been associated with human diseases [3,4]. *Listeria* spp. such as *L. innocua* are frequently employed as indicators of favorable conditions for the growth of *L. monocytogenes* [5,6].

*L. monocytogenes* is a Gram-positive, rod-shaped, and facultative anaerobic bacterium [1,7]. This bacterium is ubiquitous in the agricultural environment and has been found in soil, water, plants, and animals. *L. monocytogenes* has extraordinary adaptive abilities and can survive in extreme conditions that typically function to inhibit the proliferation of bacteria, such as the temperature range from 45 °C to −1.5 °C, which allows growth at refrigeration temperatures [8,9]. In addition, this bacterium has the ability to proliferate over a wide pH range (4.4–9.6), low water activity (aw < 0.90), and high salt concentration (even 20%) and in the presence of disinfectants and preservatives [9,10]. Furthermore, *L. monocytogenes* can form biofilms on both living and inanimate surfaces, protected from disinfectants, which further increases its ability to survive under difficult conditions [11]. However, it is unable to withstand pasteurization temperatures, which is why contamination in food processing plants typically occurs during post-pasteurization processes [11,12,13]. This causes *L. monocytogenes* to persist in food production plants, enter the food chain, and lead to contamination of many food products [13], such as raw and processed meat [8,14], fish, unpasteurized milk and dairy products, raw fruit and vegetables and ready-to-eat products [15,16,17]. The main route of spread of *L. monocytogenes* to humans is the intake of highly contaminated food [18], with foodborne transmission accounting for as much as 99% [4,13,19]. From these points of view, managing *L. monocytogenes* during industrial food processing presents significant challenges [19,20].

*L. monocytogenes* is the causative agent of a serious foodborne illness called listeriosis [17,21]. The symptoms of listeriosis are influenced by many factors, such as infectious dose, age, immune status of the consumer, and strain virulence [21,22]. This disease can manifest in invasive or non-invasive forms [23]. For individuals with a healthy immune system, a non-invasive form of listeriosis develops [22,24] and can lead to mild gastroenteritis symptoms such as nausea, vomiting, diarrhea, and fever [2,24]. In immunocompetent people, diseases are self-limiting, often resolved without the need for medical attention, resulting in undiagnosed cases and underreporting [8]. An invasive form of listeriosis develops in susceptible people, such as pregnant women, newborns, and the elderly, as well as people with weakened immune systems, such as those who have received organ transplants. In these groups, listeriosis can lead to life-threatening symptoms such as sepsis, bacterial meningitis [14], or brain infections [25]. In pregnant women, it can cause mother-to-fetus infections, resulting in spontaneous abortion, stillbirth, or premature birth [11,25,26]. Generally, listeriosis causes high hospitalization rates (95%) [26] and high mortality rates, reaching 20–30% [27]. In contrast, infection with other common foodborne illnesses, such as campylobacteriosis and salmonellosis, seldom leads to deaths [26,27,28,29,30]. For this reason, *L. monocytogenes* has been classified as one of the four foodborne pathogens considered the most significant threat to public health by the World Health Organization (WHO) since the early 1980s [31,32].

*L. monocytogenes* has several molecular mechanisms that allow it to adapt throughout the various stages of its pathogenic lifecycle [33]. The transition to an intracellular lifestyle for pathogens involves an upregulation of gene products that facilitate cell-to-cell spread and enhance bacterial replication within the host’s cytosol [17,34]. After ingestion of contaminated food, *L. monocytogenes* must survive exposure to tough conditions in the host system, e.g., high acidity and bile salts. Surviving these conditions is crucial to the pathogenesis of this bacteria. Moreover, *L. monocytogenes* has the ability to cross three critical barriers in the human body: the intestinal epithelium, the blood–brain barrier, and the placenta [35]. After binding to the epithelial cells of the gastrointestinal tract with the help of the internalin proteins InlA and InlB (encoded by *inlA* and *inlB*), *L. monocytogenes* is engulfed by macrophages within an initial phagosomal vacuole [8]. Once inside, *L. monocytogenes* escapes from the membrane-bound vacuole by secreting a pore-forming cytolysin called listeriolysin O (LLO) (encoded by *hly*), along with two phospholipases (encoded by *plcA* and *plcB*) that collectively dismantle the phagosome where the bacteria reside. Inside the cytosol of the host cell, the bacteria replicate by utilizing nutrients sourced from the host [33]. *L. monocytogenes* then moves through the cytoplasm and invades neighboring cells, utilizing actin polymerization as a means of motility, which is directed by its surface protein actin assembly-inducing protein (ActA) (encoded by *actA*) [33]. All gene products involved in bacterial invasion, cytosolic entry, growth, intracellular movement, and spread to neighboring cells are regulated by the transcriptional regulator PrfA [34]. The most important virulence determinants are clustered in the chromosome in pathogenicity islands, such as *Listeria* pathogenicity island 1 (LIPI-1), LIPI-3 and LIPI-4 [8,35].

The incidence of listeriosis in Europe has been rising in recent years [36,37,38]. Significant shifts in food production, processing, and distribution practices, along with the growing reliance on refrigeration as a primary method of preservation; alterations in dietary habits, especially an increased consumption of ready-to-eat (RTE) foods [38,39]; and demographic changes, such as a rise in the population at higher of for the disease because of aging are all proposed as potential factors contributing to the emergence of human foodborne listeriosis [40].

Due to these factors, many countries, including the USA, enforce a strict zero-tolerance policy regarding the presence of *L. monocytogenes* in RTE foods. In contrast, the European Regulation on Microbiological Criteria for Foodstuffs No. 2073/2005 sets forth two different standards for *L. monocytogenes* in RTE foods based on considerations such as pH, water activity, and bacterial growth potential. One standard applies to RTE foods for infants and special medical purposes, banning *L. monocytogenes* in 25 g of food, while the other allows up to 100 cfu/g in RTE foods not intended for those purposes throughout the product’s shelf life [41]. This highlights the need for the industry to use precise and reliable methods to detect *L. monocytogenes* in food products [2].

In recent times, several techniques for detecting *Listeria* spp., including *L. monocytogenes*, in food have been developed. Advances in diagnostic technologies allow for more accurate and faster detection of *L. monocytogenes* bacteria. This means that more cases are identified, which increases the number of reported cases [42]. These methods can be divided into two main groups: conventional methods, such as culture-based techniques, and alternative methods [2,4]. Food laboratories commonly use conventional microbiological techniques for detecting *Listeria* spp., following guidelines from organizations such as the U.S. Food and Drug Administration (FDA) and the International Organization for Standardization (ISO). These methods include standards such as EN ISO 11290-1:2017 [43] and EN ISO 11290-2:2017 [44], which involve steps such as pre-enrichment and enrichment in selective or differential media, such as *Listeria* Agar Ottaviani and Agosti (ALOA), Oxford, or PALCAM, followed by biochemical analysis, e.g., β-hemolysis test [4,12]. Traditional culture-dependent methods remain the gold standard; however, they are time-intensive and significantly dependent on the phenotype that is subject to different environmental conditions [2]. Consequently, faster alternative methods have emerged, employing various approaches. These include molecular techniques, such as polymerase chain reaction (PCR) and its variations, such as multiplex PCR and real-time PCR (RT-PCR), based on the detection of species-specific DNA sequences, including 16S rRNA genes and virulence genes, as well as immunological methods, which rely on antigen–antibody interactions, such as enzyme-linked immunosorbent assay (ELISA), along with microarrays and biosensors [1].

Due to the fact that there are many strains of *L. monocytogenes*, further subtyping is necessary. Conventional subtyping methods include serotyping. *L. monocytogenes* strains can be recognized by specific combinations of somatic (O) and flagellar (H) antigens present on their cell surfaces [35]. As of today, *L. monocytogenes* consists of four lineages (I, II, III, and IV) and 14 serotypes. Lineage I includes strains classified under serotypes 1/2b, 3b, 4b, 4d, 4e, 4h, and 7, while lineage II consists of strains belonging to serotypes 1/2a, 1/2c, 3a, and 3c. Lineage III encompasses strains from serotypes 4a and some 4b and 4c strains, whereas some 4a, 4b, and 4c strains have been characterized into lineage IV [8]. However, 95% of isolates obtained from food and clinical cases of listeriosis in humans belong to three of these serotypes: 4b, 1/2a and 1/2b. This indicates that certain serotypes may exhibit higher virulence or a greater ability to adapt to the human host [45,46]. More interestingly, serotype 1/2a is the most commonly isolated from food, while serotype 4b is primarily responsible for listeriosis infections [45,47]. For the serotyping of *L. monocytogenes*, both standard agglutination methods with mono-/polyvalent antisera [16] and PCR-based genoserotyping methods can be used [43]. Other *L. monocytogenes* subtyping methods include methods based on the use of restriction enzymes, such as pulsed-field gel electrophoresis (PFGE), and subtyping methods based on PCR, such as random amplified polymorphic DNA (RAPD) or multilocus sequence typing (MLST) [2]. Despite the great diversity of virulence of *L. monocytogenes* serotypes, in the European Union, including Poland, there is no obligation to identify the serotypes of this bacterium [46].

## 2. Resistance to Stresses Associated with Food Production

As mentioned above, *L. monocytogenes*, as a non-spore-forming pathogen, has remarkable adaptive abilities to food stresses [10]. The key role in the stress response is played by the two-component systems, such as LiaRS, LisRK, CesRK, AgrCA, and VirRS, controlled by *σB* (SigB), an alternative sigma factor that controls the general stress response in *L. monocytogenes* [47]. *L. monocytogenes*’ exceptional ability to withstand stressful conditions makes it a serious challenge in food processing and an ideal model organism for studying resistance mechanisms to stress factors found in food and food production environments. In this review, we will touch on the mechanisms of resistance to common stresses occurring in food processing plants, such as high and low temperatures, acidic pH, and osmolarity, and we will primarily concentrate on the genetic factors and mechanisms underlying *L. monocytogenes*’ susceptibility and resistance to less-studied stress conditions, such as bacteriocins.

Thermal treatment is a widely used and effective method for eliminating foodborne pathogens, as most are highly sensitive to elevated temperatures. However, it is important to note that the effectiveness of thermal treatments against *L. monocytogenes* is reduced due to its ability to survive and reproduce within a broad temperature range, from −0.4 °C to 45 °C [48]. The heat resistance of *L. monocytogenes* is influenced by various factors, including prior environmental stresses, the food matrix, and internal characteristics such as serotypes. Previous studies [49,50] have shown that strains of serotype 1/2a, the most prevalent in food, demonstrated relatively low heat tolerance, whereas serotype 4b strains, which are linked to human listeriosis, showed significant variability in their response to heat. The highest heat tolerance was observed in serotype 7 strain [49,51]. At the molecular level, *L. monocytogenes* reacts to heat treatment by expressing genes associated with specific heat shock responses, such as the production of heat shock proteins (HSPs) [52]. Class I heat shock genes, such as *dnaK*, *dnaJ*, *grpE*, *groEL,* and *groES*, produce heat shock proteins that act as chaperones to prevent the misfolding of proteins that can occur during environmental stress. These genes are controlled by the negative regulator HrcA [52]. Class II HSP genes encode general stress proteins that are regulated by the alternative sigma factor SigB under various growth-inhibiting conditions [53]. Additionally, class III heat shock genes encode ATP-dependent proteases, including ClpC, ClpP, and ClpE, controlled by the gene regulator CtsR. Class III is essential for degrading misfolded proteins under stress conditions such as elevated temperatures [52].

Equally dangerous seems to be the resistance of *L. monocytogenes* to low, refrigerated temperature treatment. More concerning is the observation that the population of *L. monocytogenes* remained unchanged during freezing [53]. Resistance to low temperatures is provided by several mechanisms. First of all, *L. monocytogenes* diminished metabolic functions by reducing intracellular enzyme activity to the essential minimum and modifying the expression of genes associated with the biosynthesis of nutrients, including lipids, carbohydrates, and amino acids, as well as those related to motility [54,55]. Furthermore, *L. monocytogenes* alters its membrane lipid composition by increasing the concentration of unsaturated fatty acids, thereby achieving optimal membrane fluidity for transport across the membrane [49,56]. Other adaptation mechanisms of *L. monocytogenes* to low temperatures have also been documented. One example of this mechanism is the expression of cold shock proteins called Csps [55]. Csps are small proteins, acting as chaperones, responsible for supporting essential life processes in bacteria [57]. Finally, *L. monocytogenes* increases the accumulation of cryoprotective substances such as carnitine, glycine betaine, and proline betaine [49]. Chan et al. [54] showed increased activity of *L. monocytogenes* at refrigeration temperature compared to human body temperature. The *opuCABCD* operon encodes a carnitine transporter, and *gbuC* encodes the binding protein of a glycine betaine transporter. These substances, which also function as osmolytes, have been found to play a role in osmotic stress resistance in *L. monocytogenes*. They help maintain proper turgor pressure and protect enzymes from degradation [49]. The study by Schmid et al. [57] also confirmed the involvement of Csps in the repair of DNA damaged by high concentrations of NaCl. These studies show the occurrence of simultaneous resistance to different stresses. The mechanism seems to further increase the problem of *L. monocytogenes* persistence in food processing plants.

Another challenge faced by *L. monocytogenes* in food processing is acidification, which occurs during fermentation by lactic acid bacteria (LAB). These bacteria are naturally present in food, originating from raw materials, and are also intentionally introduced as starter cultures [58]. *L. monocytogenes* employs several mechanisms to regulate its internal pH when exposed to acid stress. The main mechanism involved in the protection of bacteria against acidification stress is the glutamic acid decarboxylase (GAD) system. This system is composed of three gene products—GadA, GadB, and GadC (encoded by three genes *gadD1*, *gadD2*, and *gadD3*)—that function by converting extracellular glutamate into γ-aminobutyrate (GABA) through an enzymatic reaction [59]. This mechanism helps protect the bacteria in environments where the pH drops below 4.5. Another protective strategy involves the arginine and agmatine deiminase pathways [60]. These systems play a key role in *L. monocytogenes*’ response to extreme acidity, helping to regulate internal pH by converting arginine into ammonium through a series of reactions. The low pH adaptation systems mentioned above function simultaneously to ensure the survival and adaptation of *L. monocytogenes* under acid-stress conditions [52].

*L. monocytogenes* has developed a range of mechanisms to withstand various stressors in food environments. It can deploy multiple strategies to combat the same type of stress or use a single mechanism to adapt to different stressors [48]. Biopreservation offers an alternative control measure for improving the stability and safety of food products. It reduces the number of chemical preservatives needed and the intensity of heat treatments, both of which can negatively affect food quality [61]. Using bacteriocins as biocontrol agents is a promising approach to managing pathogens like *L. monocytogenes.* Both Gram-positive and Gram-negative bacteria are capable of producing bacteriocins. Our main interest lies in the compounds synthesized by LAB [62].

## 3. Bacteriocins

LAB are part of a broad group of Gram-positive, microaerophilic microbes known for producing lactic acid as the primary byproduct of glucose fermentation in dairy products, fermented meat products, and fermented vegetables [58,63]. LAB consists of the following genera: *Lactobacillus*, *Lactococcus*, *Pediococcus*, *Leuconostoc*, *Enterococcus*, *Streptococcus*, *Carnobacterium*, and *Weissella*, and more peripheral genera such as *Aerococcus*, *Tetragenococcus,* and *Oenococcus* [58,64]. They have been used for centuries in the production of fermented foods due to their positive impact on nutritional value, sensory qualities, and shelf life [65]. This is due to the production of antimicrobial agents, including organic acids, carbon dioxide, ethanol, hydrogen peroxide, and bacteriocins, in particular [66,67].

Bacteriocins produced by LAB are ribosomally synthesized antimicrobial peptides that exhibit bactericidal or bacteriostatic activity against strains of closely related bacteria [64]. They are regarded as narrow-spectrum bacteriocins, although some bacteriocins have a broad spectrum of activity, including species unrelated to the producer, spoilage organisms, and food-borne pathogens such as *L. monocytogenes* [64,66]. Activity against Gram-negative bacteria has been demonstrated, but typically only when the outer membrane’s integrity is compromised. Bacteriocin-producing organisms are resistant to their own antimicrobial peptides, a defense mediated by specific immunity proteins [59]. Genes responsible for the synthesis of bacteriocins are grouped into gene operons, most often located on mobile genetic elements such as bacteriocinogenic plasmid. This opens up the possibility of bacteriocin synthesis in heterologous systems [66,68].

The first classification, created by Klaenhammer [69], included four classes of bacteriocins. Today, bacteriocins are divided into three major classes based on biological activity. Bacteriocins produced by LAB are mainly classified into classes I and II. Class I bacteriocins include small, post-translationally modified peptides called lantibiotics [64]. Bacteriocins of this class, as a result of post-translational modifications, acquire unusual amino acids such as lanthionine and/or methyllanthionine. The vast majority of class I bacteriocins are produced by Gram-positive bacteria belonging to the species of LAB. The most widely studied member of group I is nisin, produced by *Lactococcus lactis*. The biosynthesis of nisin is governed by an operon composed of 11 genes, regulated by the two-component NisRK system. This system functions as part of a quorum sensing (QS) mechanism, responding to the concentration of nisin [66].

Class II bacteriocins are small (<10 kDa), thermostable, unmodified peptides. The characteristic feature of this class of bacteriocins is their bactericidal activity against the pathogen *L. monocytogenes*. For this reason, class II bacteriocins are often called listericidal bacteriocins [70]. The structure of peptides of class IIa can be divided into two different regions separated by a flexible hinge. The cationic N-terminal region contains two cysteine residues joined by a disulfide bridge and a conserved YGNGVXC motif characteristic of bacteriocins of this class [71]. The C-terminus is less conserved. Both regions of the bacteriocin contribute to its bactericidal activity: the N-terminus is involved in interacting with the target cell, while the C-terminus determines the specificity of the target cell [71]. This class can be further divided into a few subclasses, namely subclass IIa (pediocin-like bacteriocins), subclass IIb (dipeptide bacteriocins), and subclass IIc (circular bacteriocins). The most well-known representative of class II bacteriocins is pediocin PA-1, which belongs to subclass IIa. Its gene operon is plasmid-encoded and consists of four genes: the structural gene *pedA*, the immunity gene *pedB*, and two additional genes, *pedC* and *pedD*, which encode an ABC transporter and an accessory protein, respectively [72]. Like nisin, pediocin PA-1 requires a specific receptor on the surface of the target cell for its bactericidal activity.

Class III bacteriocins are large, heat-sensitive protein molecules with molecular weights exceeding 30 kDa. Unlike other bacteriocins that remain stable at high temperatures, class III bacteriocins are prone to heat denaturation, which restricts their application in certain food processing scenarios. These bacteriocins function primarily by enzymatically degrading the cell walls of target bacteria, resulting in cell lysis. One example of a class III bacteriocin is helveticin J, which is produced by *Lactobacillus helveticus* [73]. The classification of bacteriocins is presented in Table 1.

Bacteriocins produced by LAB are of particular interest because many have a long history of safe use, and most LAB species are recognized as safe under the GRAS (Generally Recognized as Safe) and QPS (Qualified Presumption of Safety) classifications [66]. Bacteriocins can be utilized as natural preservatives, and with the increasing consumer preference for more natural products, they offer a promising alternative to chemical preservatives [72,75]. Bacteriocins possess numerous qualities that make them appealing as biopreservatives in food products. Defined as natural antimicrobial agents, they align with the increasing consumer preference for natural and minimally processed foods. Unlike many chemical preservatives, bacteriocins are non-toxic to humans; as protein compounds, they are degraded by proteolytic enzymes in the digestive system into simple, harmless components that are easily absorbed and metabolized. Due to their decomposition, they do not accumulate in the natural environment. As a result, they do not alter the composition of the gut microbiota and are non-cytotoxic and non-carcinogenic [74,78].

One significant advantage of bacteriocins over chemical preservatives or traditional sanitization methods is their selectivity and specific spectrum of action. They often display bactericidal activity against foodborne pathogens such as *L. monocytogenes*, *Staphylococcus aureus*, and *Clostridium* spp., while generally remaining inactive against starter cultures used in the fermentation of foods and probiotic microorganisms [71,72,73,75,79].

## 4. Mechanisms of Bacteriocin’s Action

Bacteriocins exhibit both bacteriostatic and bactericidal effects on bacterial cells. The mechanisms underlying the inhibitory actions of bacteriocins from LAB toward target cells exhibit a substantial diversity. The cationic nature of bacteriocins facilitates their docking onto anionic elements of the bacterial cell surface and interaction with the bacterial cell membrane or a specific membrane receptor [76,80]. In the next stage, bacteriocins induce permeabilization of the target bacterial cell membrane, which disrupts the proton motive force and depletes intracellular ATP, causing leakage of cell contents and, ultimately, cell death [77]. To date, seven receptors have been identified. The first to be identified and the most studied is lipid II. Lipid II, located in the cytoplasmic membrane, is pivotal in constructing the cell wall by transporting peptidoglycan monomers from inside to outside [77,81]. Lipid II is a crucial precursor targeted by nisin. Binding to lipid II, nisin disrupts cell wall synthesis and forms membrane pores, leading to bacterial cell death. Nisin also acts by blocking lipid II, inhibiting cell wall synthesis [81,82].

The second receptor is the mannose-specific phosphotransferase system (Man-PTS), the primary mannose transport system in Firmicutes and Gammaproteobacteria [80]. Man-PTS generally includes three proteins: enzyme I (EI), histidine phosphocarrier protein (HPr), and enzyme II (EII). EI and HPr, both cytoplasmic proteins, facilitate the transfer of a phosphate group to EII. EII comprises four subunits: IIA, IIB, IIC, and IID. IIA and IIB are found in the cytoplasm, while IIC and IID create a membrane-bound complex that allows sugar molecules to enter the cell [83,84]. The membrane subunits IIC and IID of Man-PTS are vital for the bactericidal effect of class IIa bacteriocins like pediocin PA-1 [66,83]. By binding to Man-PTS, pediocin-like bacteriocins cause cell membrane permeabilization, disrupt the proton motive force, and deplete ATP and intracellular substrates. This terminates all cellular biosynthesis, ultimately leading to cell death [84,85].

Other receptors include undecaprenyl pyrophosphate phosphatase UppP, the APC family amino acid transporter, CorC protein, ABC maltose transporter, and zinc-dependent metallopeptidase YvjB [80]. Receptor-dependent bacteriocins have a distinct, narrow range of activity, making them highly appealing for specific applications. Consequently, investigating receptor identification and bacteriocin-receptor interaction mechanisms is of paramount importance [80].

## 5. The Usefulness of Bacteriocins in Eliminating *L. monocytogenes* from Food

Bacteriocins produced by LAB are excellent for preserving food at risk of *L. monocytogenes* contamination. Especially promising is their potential to enhance the microbiological safety of minimally processed foods like dairy, meat, and various fruits and vegetables. Research has demonstrated that class IIa bacteriocins effectively reduce *L. monocytogenes* levels in many food products, making them a valuable tool in ensuring food safety [80,86].

Bacteriocins from different species of LAB have shown both bactericidal and bacteriostatic effects on *L. monocytogenes*. For instance, a starter culture of *Pediococcus acidilactici* producing pediocin PA-1 reduced *L. monocytogenes* in poultry sausages by up to 2.6 log cfu/g [87]. Similarly, pediocin PA-1 decreased *L. monocytogenes* in ripened cheese by up to 3 log cfu/g [88]. In cold-smoked, vacuum-packed salmon, bactericidal effects were noted for bacteriocins produced by *Carnobacterium* species. Piscicocin VIa and VIb, both produced by *Carnobacterium piscicola* V1, showed bactericidal activity, while divercin V41 from *Carnobacterium divergens* V41 demonstrated bacteriostatic activity [89]. Piscicolin 126, from *Carnobacterium piscicola* JG126, permanently reduced *L. monocytogenes* to undetectable levels in ham [80]. Additionally, saccacin P from *Lactobacillus sakei* Lb790 inhibited *L. monocytogenes* in vacuum-packed chicken slices, remaining stable on cold cuts for 4 weeks [90]; it also had bactericidal activity in cold-smoked salmon [91]. Enterocin A, produced by *Enterococcus faecium* strain CTC492, reduced *L. monocytogenes* by 1 log in dry fermented sausage and showed bactericidal activity when combined with enterocin B [90,92]. These studies demonstrate that bacteriocins, especially those from class IIa, are highly effective against *L. monocytogenes*, making them a powerful tool in food safety.

Bacteriocins can be incorporated into food through several methods and forms. One approach is to inoculate the food with a bacteriocin-producing strain [78]. Another option is to add purified bacteriocins as food additives [72]. Additionally, food can be processed using ingredients previously fermented with bacteriocin-producing strains. Bacteriocins may also serve as a component in bioactive packaging [80,93].

The use of bacteriocins in food is one of the dynamically developing areas of biopreservation. However, the number of bacteriocins widely used in the food industry remains limited. Despite the discovery and study of numerous bacteriocins, only a few have been commercially implemented. To date, only two bacteriocins have been approved for widespread use in the food industry: nisin, marketed as Nisaplin^®^ with the active ingredient nisin A, and pediocin PA-1, sold under the brand name ALTA™2431. Therefore, most research in this area has focused on specific bacteriocins, such as nisin and some bacteriocins of class IIa [93,94].

## 6. Development of Bacteriocin Resistance

For a bacteriocin to be approved for general use, it must meet several key requirements. The first aspect is the efficiency of using bacteriocins. The bacteriocin must demonstrate a narrow spectrum of action and strong antimicrobial activity against target pathogens and, at the same time, should not affect technological microorganisms such as starter cultures or probiotics. The second aspect is safety. It must be non-toxic to humans and animals and not cause adverse effects [95,96]. The third and most important aspect is the potential risks of resistance development [97,98]. The development of highly tolerant or resistant strains is a significant concern, as it undermines the effectiveness of bacteriocins as biopreservatives [97]. The global problem of resistant foodborne pathogens is exacerbated by the international trade of raw and processed foods. This facilitates the spread of resistant strains across borders, making it a significant public health concern [72].

Numerous studies have explored the susceptibility of pathogenic bacteria, such as *L. monocytogenes*, to specific bacteriocins. However, comparing these studies can be challenging due to varying experimental methods and terminology. In the literature, bacteriocin resistance is often not clearly defined, and there is no consensus among researchers regarding what constitutes high, moderate, or low resistance levels [80]. A key element in susceptibility tests is the bacteriocin solution itself. Some studies use purified preparations, such as nisin, measured in milligrams or international units (IU), while others use fermentates from producer organisms, such as pediocin PA-1, measured in activity units (AU) [82]. In the study by Gravesen et al. [98], the resistance of *L. monocytogenes* to nisin was defined as a 10-fold increase in the minimum inhibitory concentration (MIC). If a pathogen could grow at concentrations higher than MIC, it was deemed resistant to the bacteriocin.

Microorganisms exhibit different types of resistance to antimicrobials: innate, apparent, or acquired. Innate resistance is genetically controlled and naturally associated with the organism. Differences in resistance among species and strains under identical conditions are typically due to innate factors. Mechanisms include cellular barriers (such as teichoic acids in Gram-positive bacteria), efflux mechanisms (pumping out compounds), lack of biochemical targets, and antimicrobial inactivation by enzymes. Natural resistance to nisin in *L. monocytogenes* occurs at frequencies ranging from less than 10^−9^ to less than 10^−5^ [99]. In contrast, resistance to class IIa bacteriocins has been observed in 1 to 8% of wild-type strains tested [72]. Apparent resistance relates to assay or application conditions. Susceptibility varies based on application settings and interacting stress conditions (such as high temperatures, low pH, or high pressure), which can affect resistance. Acquired resistance results from genetic changes via mutations or acquiring genetic material from plasmids, altering the microbial cell’s response to antimicrobials. Acquired resistance to bacteriocins in *L. monocytogenes* may be the result of factors resulting from human practices. Overuse of antimicrobial compounds in food preservation and other branches, such as agriculture, veterinary, cosmetics, and medicine, may create selection pressure on bacteria, encouraging the development of resistant strains. Furthermore, the use of sublethal concentrations of bacteriocins allows bacteria to survive and adapt, enhancing resistance [61]. Various genetic loci have been linked to these types of resistance [100,101]. Understanding these genes’ roles in bacteriocin resistance is crucial for optimizing bacteriocin use and continues to attract research interest [102]. The main mechanisms and genetic determinants associated with *L. monocytogenes* resistance to LAB bacteriocins are presented in Table 2.

### 6.1. Resistance via Changes in Receptor Expression

As previously noted, for many bacteriocins to be effective in eliminating bacteria, they need to bind to specific receptors on the target cell’s surface. These receptors act as docking sites, allowing the bacteriocin to attach and exert its lethal effects. Without the presence of these specific receptors, the bacteriocins cannot effectively interact with the bacterial cell, rendering them less effective or even ineffective. The phenomenon of resistance to nisin, a class I bacteriocin, was described by Gravesen et al. [81]. In this study, the researchers aimed to identify loci responsible for nisin resistance in *L. monocytogenes*. Their analysis revealed that the nisin-resistant mutant exhibited increased expression of a protein with strong homology to the glycosyltransferase domain of high-molecular-weight penicillin-binding proteins (PBPs). These membrane-bound proteins are crucial for cell wall biosynthesis through peptidoglycan chain extension and cross-linking. It was speculated that the increased expression and production of this protein might partially shield lipid II in the plasma membrane, thus reducing nisin’s efficacy by impeding its access to the binding site. The other authors observed a similar effect [81].

Resistance to subclass IIa bacteriocins in *L. monocytogenes* often involves changes in receptor expression. This resistance can arise through either the reduced expression or even loss of the Man-PTS system, a key element involved in sugar uptake. According to Vadyvaloo et al. [103], wild-type strains demonstrated faster growth in the presence of glucose, whereas the class IIa bacteriocin-resistant strains exhibited quicker growth in the absence of glucose. The variations in growth between the wild-type and resistant mutants on different carbohydrates suggest that the sugar metabolism pathways might be altered in the resistant strains. The man-PTS system is encoded by the *mptACD* operon. The level of *mptACD* expression has been shown to correlate with the level of bacteriocin sensitivity [61]. Survival of bacteria under stress conditions requires rapid changes in gene expression, which are controlled by the association of different alternative sigma factors. Previous studies reviewed by Bastos et al. [111] have identified four types of sigma factors in *L. monocytogenes*: *σB* (SigB), *σH*, RpoD, and RpoN.

The study by Robichon et al. [106] linked resistance to subclass IIa bacteriocins to the regulatory gene *rpoN*, which encodes the alternative sigma factor σ54. It was proposed that the transcription factor σ54 might regulate the expression of the Man-PTS receptor, as mutants of *L. monocytogenes* lacking *rpoN* [106] showed resistance to mesentericin Y105 (produced by *Leuconostoc mesenteroides*) and related subclass IIa bacteriocins. σ54 is known to be involved in the regulation of PTS expression. The study by Dalet et al. [112] discovered that the mannose family activator ManR and the PTS permease are essential for *L. monocytogenes* sensitivity to mesentericin Y105. σ54 activates the *mptACD* operon (*mptA*, *mptC*, and *mptD* genes), which codes for EIIAB, EIIC, and EIID in *L. monocytogenes*, alongside ManR, the transcriptional activator for *σ54* [112]. Disruptions in the *mptA* or *mptD* genes led to resistance, and further in-frame deletions in the *mptD* gene were also linked to mesentericin Y105 resistance, suggesting that a specific domain of the MptD subunit is involved in target recognition by the bacteriocin. These findings were the first to suggest that EIIMan could serve as a receptor for subclass IIa bacteriocins [106]. Mutations in *rpoN* led to the loss of *mptACD* expression, resulting in resistance to mesentericin Y105 [112]. Similarly, mutations in ManR (encoded by *manR*) rendered the cells resistant to subclass IIa bacteriocins [108]. Both regulatory proteins, RpoN and ManR, are crucial for the active transcription of genes encoding the subclass IIa bacteriocin receptor.

In the works of Gravesen et al. [113] and Ramnath et al. [114], two different changes in PTS expression were correlated with the development of resistance to class IIa bacteriocins in *L. monocytogenes*. In the study by Ramnath et al. [109], a mutant of *L. monocytogenes* resistant to leucocin A (produced by *Leuconostoc gelidum*) was found to lack the IIAB subunit of the mannose PTS permease. Conversely, Gravesen et al. [113] identified that *L. monocytogenes* mutants resistant to pediocin PA-1 exhibited overexpression of the β-glucoside PTS permease. Gravesen et al. [108] examined eight mutants of *L. monocytogenes* and found that all high-level resistance strains showed increased expression of two putative β-glucoside-specific PTS genes. Additionally, these strains failed to synthesize the MptA subunit of the mannose-specific PTS, EIIMan. This suggests that spontaneous resistance to class IIa bacteriocins in *L. monocytogenes* arises through a single mechanism (downregulation of the Man-PTS gene) that causes two distinct changes in PTS expression. However, disruption of these genes in the resistant mutant did not confer sensitivity to pediocin. This indicates that increased expression of β-glucoside PTS permease is not a direct cause of resistance but is probably a regulatory consequence of acquired resistance through the abolition of *mptACD* expression [103]. Preventing *mptACD* expression directly confers resistance. Expression of *mptACD* could be prevented by mutations in all the factors mentioned earlier, e.g., *rpoN*, *manR,* or *mptACD* [112].

Since the IIC and IID subunits of the *mptACD* operon are likely membrane-bound, they were hypothesized to be potential targets for class IIa bacteriocins. When the *mptACD* operon of *L. monocytogenes* was expressed in an insensitive species like *Lactococcus lactis*, this strain became sensitive to various class IIa bacteriocins. Individual expression of each gene from the *mptACD* operon in *Lactococcus lactis* revealed that expressing *mptC* alone was enough to confer sensitivity. Therefore, the IIC subunit was proposed as the target molecule for class IIa bacteriocins [114].

Both Tessema et al. [102] and Kjos et al. [70] confirmed findings by Gravesen et al. [108] that the primary mechanism of resistance in *L. monocytogenes* involves the downregulation of Man-PTS gene expression. This downregulation, caused by the lack of a functional *mptACD*, reduces or eliminates receptor proteins, leading to high resistance levels. Furthermore, both studies identified additional resistance mechanisms. Tessema et al [99] found that resistant strains exhibited changes in carbon catabolite control, likely mediated by *mptACD*, along with cell envelope modifications and bacteriocin efflux through the TAT system, contributing to the resistance in sakacin P-resistant strains. Kjos et al. [70] noted that cells with intermediate resistance had high Man-PTS gene expression, similar to wild-type cells. This was linked to metabolic shifts and suggested changes in cell surface properties that affect bacteriocin-receptor interactions.

### 6.2. Resistance Due to Changes in the Cell Envelope

#### 6.2.1. Resistance Due to Changes in the Cell Wall

Gram-positive bacteria, including the pathogenic *L. monocytogenes*, are characterized by the presence of peptidoglycan in their cell walls. This crucial component is a polymer made of sugars and amino acids, forming a protective layer outside the plasma membrane, which provides structural strength and rigidity. Peptidoglycan contains two types of anionic polymers: teichoic acids (TAs), which are covalently attached to the peptidoglycan, and lipoteichoic acids (LTAs), which consist of polyphosphoglycerol substituted with a D-alanyl (D-Ala) ester, anchored in the membrane by their glycolipid component [115]. Those components cause the cell wall to carry an anionic charge due to deprotonated phosphate groups [82]. Thus, altering the surface charge of the target cell wall emerges as a critical defense strategy against cationic bacteriocins. Changing the bacterial surface charge is likely to impact the initial electrostatic interaction between the peptide and the membrane, which is crucial for pore formation [72].

The *dlt* operon, consisting of four genes (*dltA*, *dltB*, *dltC,* and *dltD*), was characterized in the study by Abachin et al. [115]. These genes are responsible for incorporating D-alanine residues into cell-wall-associated LTAs. A mutant deficient in D-alanine, created by inactivating the *dltA* gene (which encodes a cytoplasmic D-alanine-D-alanyl carrier protein ligase), exhibited increased susceptibility to cationic peptides such as nisin. The study by Vadyvaloo et al. [103] also emphasized that decreasing the negative charge of the cell wall contributes to resistance. This reduction is achieved by increasing the D-alanine content in teichoic acids, which lowers the cell wall’s negative charge and, consequently, its susceptibility to bacteriocins. Gravesen et al. [108] demonstrated that a notable decrease in the expression of *dltA*, *dal*, and *dat* genes can reduce the availability of D-alanine. This shortage affects the incorporation of D-alanine into LTAs, TAs, and peptidoglycan.

Collins et al. [100] identified the *lmo1967* locus in *L. monocytogenes* as key for innate nisin resistance. This locus is a homolog of the tellurite resistance gene *telA*. Mutant analysis revealed that mutants were four times more susceptible to nisin and twice as susceptible to certain antibiotics and tellurite. This study was the first to associate the *telA* gene with resistance to antimicrobials targeting the cell envelope.

#### 6.2.2. Changes in the Fatty Acid Composition of the Cell Membrane

Research has consistently demonstrated that alterations in the surface charge of the cell wall significantly contribute to *L. monocytogenes*’ resistance to cationic bacteriocins. Additionally, it is hypothesized that this bacterium might adopt various cell membrane modifications to further bolster its resistance [116].

In the study by Vadyvaloo et al. [117], the researchers focused on the first mechanism involving cell membranes: the alteration of membrane fluidity. The study examined the relationship between leucocin A, a class IIa bacteriocin, and the composition of the major phospholipid, phosphatidylglycerol (PG), in both susceptible and resistant strains of *L. monocytogenes.* The analysis revealed that resistant strains had an increased ratio of unsaturated to saturated and short to long PG acyl chains. This shift towards PGs with shorter, unsaturated acyl chains increased membrane fluidity. This decrease may hinder the insertion of class IIa bacteriocins into the membrane and affect the stability of the pore complex, contributing to resistance. Membrane adaptation is likely just one of several mechanisms involved in resistance, and other mechanisms are necessary for complete resistance. Ming and Daeschel [118] also observed that a nisin-resistant mutant of *L. monocytogenes* had a higher proportion of straight-chain fatty acids compared to its parent strain, which exhibited a greater amount of branched-chain fatty acids. In addition, the resistant strain showed less phosphatidylglycerol and cardiolipin than the wild-type [118]. In *L. monocytogenes* ATCC 700302, which is resistant to nisin, researchers observed comparable alterations in membrane fatty acid composition, including an increase in long-chain fatty acids, a decrease in short-chain fatty acids, and a reduction in the C15/C17 ratio [111]. The observed changes in fatty acid composition suggest a decrease in cytoplasmic membrane fluidity. This increased stiffness likely impedes nisin from penetrating the membrane.

Vadyvaloo et al. [103] explored another mechanism behind bacteriocin resistance in *L. monocytogenes*: the alteration of membrane surface charge. The cationic nature of peptides allows them to interact with negatively charged cell surfaces, leading to membrane permeabilization [72,82]. Altering the bacterial surface charge can thus impact the electrostatic interaction between the peptide and membrane [108]. One method of charge modulation is through L-lysinylation of TA and LTAs in the cell wall. Vadyvaloo et al. [103] found that highly resistant strains showed increased lysinylation of membrane phospholipids. Normally, phospholipids such as L-lysyl-PG and L-lysyl-cardiolipin are negatively charged, but adding L-lysine to produce lysylphosphatidylglycerol, a basic phospholipid [118], changes their net charge to positive, reducing the anionic properties of cell permeability barriers and thus decreasing susceptibility to cationic antimicrobial compounds.

Verheul et al. [104] demonstrated that modifications in the cytoplasmic membrane composition could contribute to lantibiotic resistance in *L. monocytogenes*. In their study, a nisin-resistant mutant of *L. monocytogenes* Scott A, developed through exposure to increasing concentrations of nisin, showed reduced diphosphatidylglycerol and increased phosphatidylglycerol production compared to the parent strain. Nisin penetrates diphosphatidylglycerol lipid monolayers more effectively than other lipids, including phosphatidylglycerol; thus, the resistance in the mutant strain was linked to the decreased diphosphatidylglycerol content in its cytoplasmic membrane. Additionally, factors such as the zwitterionic phosphatidylethanolamine content in the phospholipids of *L. monocytogenes* can influence the net surface charge [119].

The lysinylation process requires the MprF protein, a membrane-localized lysylphosphatidylglycerol synthetase encoded by the *mprF* gene [77]. When there is a mutation in the *mprF* gene, it interferes with the incorporation of lysine into membrane phospholipids. This results in an increased negative charge of the cell envelope, making the bacteria more vulnerable to bacteriocins and other cationic antimicrobial peptides (CAMPs) [120].

The study by Mandin et al. [109] underscored the importance of VirR, a response regulator in the two-component signal transduction system (2CS) VirRS, in *L. monocytogenes*. VirRS system proteins are encoded by *vir* operon that includes a response regulator gene (*virR*) and a histidine kinase gene (*virS*). VirR, as revealed through transcriptomic approaches, positively regulates the transcription of 12 genes, including *mprF* and *dltACD* operon, both involved in defense against bacteriocin. It has been demonstrated that inactivating VirR increases bacterial susceptibility to bacteriocins [120]. Additionally, the VirRS system in *L. monocytogenes* is influenced by alternative sigma factors, SigB, which contributes to resistance to bacteriocins.

Several other 2CSs play a role in bacteriocin resistance. One notable example is the AnrAB, an ATP-binding cassette (ABC) transporter in *L. monocytogenes*, which not only contributes to innate bacteriocin resistance but also provides protection against bacitracin and beta-lactam antibiotics [121]. After *dltA* and *mprF*, *anrB* is identified as the third VirRS-regulated locus in *L. monocytogenes* linked to nisin resistance [109]. Additionally, expression studies have shown that AnrAB is regulated by RpoN [122]. It is hypothesized that AnrAB, VirRS, and Lmo1746-Lmo1747 form an antimicrobial sensing and detoxification system similar to the VraDE-BraSR-BraDE circuit in *Staphylococcus aureus* [110]. The genes *lmo1746-lmo1747*, also known as *virAB*, encode a putative ABC transporter crucial for VirR activity. The expression of *virAB* is essential for nisin resistance [122]. Cotter et al. [123] and Bergholz et al. [110] described another significant example, the LisRK two-component system. This system in *L. monocytogenes* not only helps the bacterium respond to acidic and oxidative stress but also plays a crucial role in nisin resistance and the pathogen’s inherent resistance to cephalosporin antibiotics.

### 6.3. Role of Cations in Resistance Against Bacteriocins

The studies by Abee et al. [124] and Crandall and Montville [119] highlight the significance of divalent cations in stabilizing the cytoplasmic membrane of nisin-resistant cells. This stabilization could involve interactions between the cations and envelope components such as negatively charged teichoic acids; however, it can also involve interfering with nisin’s binding. Abee et al. [124] found that divalent cations (Mg^2+^ and Ca^2+^) decreased the rate of potassium (K^+^) efflux from whole cells of *L. monocytogenes.* Scott A. Crandall and Montville [119] suggested that divalent and trivalent cations might inhibit the electrostatic interactions between the positively charged nisin molecules and the negatively charged phospholipid headgroups. Similar results were obtained by Kaur et al. [125]. Kaur et al. [125] found that adding divalent cations (Mg^2+^, Ca^2+^, and Mn^2+^) significantly decreased the inhibitory effects of nisin, pediocin 34, and enterocin FH99 against *L. monocytogenes*. However, when EDTA was added, the inhibitory activity was restored, indicating that divalent cations likely interfere with the initial electrostatic interaction between the positively charged bacteriocin and the negatively charged membrane phospholipids.

The study by Kaur et al. [125] also observed that resistant *L. monocytogenes* cells tended to form aggregates. This aggregation may lead to biofilm formation, serving as an additional resistance mechanism by reducing the contact surface area with bacteriocins, making it more difficult for the antimicrobial compounds to exert their effects. Biofilm formation is one of the resistance strategies of *L. monocytogenes* against bacteriocins. In response to nisin exposure, *L. monocytogenes* transitions from a planktonic (free-floating) state to a sessile organization, forming biofilms to better withstand environmental stress. Sublethal concentrations of nisin trigger the upregulation of proteins linked to biofilm formation in this species [126]. Liu et al. [127] discovered that the cell surface of pediocin-resistant *L. monocytogenes* variants exhibited increased hydrophobicity, potentially leading to greater cell aggregate formation. Likewise, Martínez and Rodríguez [128] found that nisin-resistant *L. monocytogenes* Lm41 mutant displayed higher hydrophobicity compared to its wild-type strain.

### 6.4. Cross Resistance Related to Growth Conditions

Elements such as bacterial growth phase, temperature, pH, and nutrient availability can influence resistance gene expression and overall bacterial resistance. *L. monocytogenes* demonstrates remarkable adaptability to the tough conditions in food processing environments, highlighting the importance of studying how stress conditions collectively enhance its resistance to bacteriocins. The study by Begley et al. [129] demonstrated a link between nisin resistance and the ability of bacteria to adapt to acidic environments, highlighting the crucial role of the glutamate decarboxylase (GAD) system. This system converts glutamate to γ-aminobutyrate (GABA) and carbon dioxide, facilitated by the enzymes GadD1, GadD2, and GadD3. This conversion also generates ATP, boosting the bacterial cell’s energy reserves. In the next stage, GABA is exported from the cell by the proteins GadT1 and GadT2. Research indicated that bacteria lacking the GadD1 gene struggled to survive in the presence of nisin, showing a 40% reduction in ATP levels; thus, GadD1 is proposed to play a key role in maintaining ATP levels, countering nisin’s harmful effects, and supporting bacterial survival [129]. Jydegaard et al. [130] investigated the impact of the growth phase, osmotic shock, and low-temperature shock on the resistance of *L. monocytogenes* to the bacteriocins nisin and pediocin PA-1. They found that the growth phase significantly impacted resistance, with stationary phase bacteria showing higher resistance to both bacteriocins compared to those in the exponential phase. Additionally, cultures exposed to osmotic stress (6.5% NaCl) and cold stress (5 °C for 60–80 min) exhibited increased tolerance. Research has sought to clarify this phenomenon. It is likely attributed to changes in the electrostatic interactions between bacteriocins and the cell surface when ion concentrations are higher [130]. Additionally, increased osmolarity in the culture medium can alter cell morphology, leading to modifications in the cell envelope [130].

De Martinis et al. [99] investigated the influence of salt, pH, and temperature on the effectiveness of nisin against *L. monocytogenes.* The research found that at temperatures 20 °C and 30 °C, resistance to nisin was stable regardless of pH and salt concentration. At 10 °C, nisin resistance decreased with lower pH and salt concentrations. Interestingly, low salt levels (2–3.5%) seemed to protect *L. monocytogenes* at 10 °C, confirming earlier findings by Cole et al. [130] about optimal salt concentrations for bacterial growth at low temperatures. This suggests that salt can help more nisin-resistant colonies survive in cold environments. Furthermore, the study showed that nisin resistance remained stable after repeated exposure, highlighting that relying solely on nisin as a preservative could lead to the development of stable resistant mutants. Therefore, nisin should be used as part of a multi-hurdle approach to food preservation [131]. These reports were confirmed in the study by Bergholz et al. [110]. This study examined cross-resistance in *L. monocytogenes* to nisin under salt stress and low-temperature conditions. They found that exposure to salt stress at low temperatures significantly increased the pathogen’s resistance to nisin. This stress increased the expression of genes related to nisin resistance, including the response regulator LiaRS. By constructing *liaR* deletion mutations in seven strains and exposing them to 6% NaCl, they found that wild-type strains exhibited a marked increase in nisin resistance after salt exposure [113]. Conversely, *liaR* mutants were more sensitive, showing that LiaFSR induction provides cross-protection against nisin. Additionally, LiaR-regulated genes such as *lmo1746* and *telA* contributed to this resistance. These findings suggest that environmental stresses similar to those found in foods can influence *L. monocytogenes*’ resistance to antimicrobials such as nisin, emphasizing the need to consider potential cross-protective effects when applying control measures against this pathogen [110].

### 6.5. Cross-Resistance to Multiple Bacteriocins

Using a combination of multiple bacteriocins simultaneously can help mitigate the risk of resistance in *L. monocytogenes.* Therefore, it is crucial to study the potential for cross-resistance between different bacteriocins [74,82]. The findings on cross-resistance in *L. monocytogenes* present varied outcomes [131] reported no cross-resistance between nisin (*Lactococcus lactis*), pediocin PA-1 (*Pediococcus acidilactici*) and bavaricin A (*Lactobacillus bavaricus*), whereas Crandall and Montville [119] reported that nisin resistance in *L. monocytogenes* conferred cross-resistance to pediocin PA-1. Similarly, Gravesen et al. [132] identified cross-resistance between nisin and subclass IIa bacteriocins such as pediocin PA-1 and leucocin A. Another study by Crandall and Montville [119] showed that a strain of *L. monocytogenes* exhibited cross-resistance to nisin, pediocin PA-1, and leuconocin S. Cross-resistance has been observed not only between different classes of bacteriocins but also within the same class. Crandall and Montville [119] also observed complete cross-resistance among subclass IIa bacteriocins such as pediocin PA-1, leucocin A, and carnobacteriocin B2.

## 7. Strategies to Overcome Bacteriocin Resistance

It is vital to grasp both the causes and mechanisms behind resistance development for effective bacteriocin management. Strategies are essential to minimize resistance risk while ensuring balanced bacteriocin use to maintain effectiveness. Simultaneously, monitoring bacterial populations for resistance is crucial for early detection and prompt responses [111,133]. First, inappropriate use should be limited. Bacteriocins need to be used precisely and in a controlled manner to avoid overuse, thereby reducing the pressure on bacteria to develop resistance [97]. Second, optimizing doses and schedules is crucial to maximize efficacy while minimizing the risk of selecting resistant strains. Additionally, rotating or cyclic use of different bacteriocins can reduce selection pressure, making it harder for bacteria to develop resistance [133].

In food technology, combining bacteriocins with other preservatives also can be an effective strategy [134]. By employing multiple bacteriocins with different mechanisms of action at the same time, the approach reduces the risk of resistance. Bacteria would need to develop resistance to several bacteriocins simultaneously, making it significantly more challenging for them to survive. For instance, combining nisin and pediocin PA-1 significantly lowered the occurrence of resistance development in *L. monocytogenes* [108]. Moreover, the combined use of bacteriocins significantly reduced MIC, effectively lowering the required inhibitory dosage [77]. Additionally, a multiple-hurdle preservation strategy, combining bacteriocins with other methods, can reduce selection pressure on bacteria [130]. Kaur et al. [125] discovered that *L. monocytogenes* mutants resistant to nisin, pediocin 34, and enterocin FH99 did not gain resistance to low pH, sodium chloride, or sodium nitrite; instead, these mutants were as sensitive or even more sensitive than the wild-type strain. Bergholz et al. [110] indicated that salt stress at low temperatures may offer cross-protection to *L. monocytogenes* against nisin. Therefore, it is important to consider the potential for cross-protective effects when utilizing various hurdle technologies for food preservation.

Other approaches involve developing new bacteriocins with unique mechanisms [111,133]. This includes creating genetically engineered bacteriocins with improved stability, greater efficacy, and a lower likelihood of inducing resistance [77]. For example, nisin A has been modified to nisin V through a single amino acid substitution, resulting in increased efficacy against *L. monocytogenes* compared to nisin A [135]. Considering all approaches, bioengineering remains one of the most promising methods for enhancing the antimicrobial activity of bacteriocins [136].

Monitoring and surveillance of resistance are critical. Regular checks on bacterial populations for resistance to bacteriocins allow for early detection and rapid response. Understanding the mechanisms and genetic determinants of resistance is vital for effective monitoring. To sum up, a holistic approach is necessary to minimize bacterial resistance to bacteriocins. Collaboration between scientists, the food industry, and healthcare is essential for managing and maintaining the effectiveness of bacteriocins in combating pathogenic bacteria [133].

## 8. Conclusions

*L. monocytogenes* is a formidable foodborne pathogen due to its ability to adapt to harsh food processing conditions and its persistence in food products. Bacteriocins, particularly those produced by LAB, show promise as biocontrol agents to enhance food safety by eliminating *L. monocytogenes* [137]. However, increasing resistance to these bacteriocins poses a significant challenge. Research on resistance mechanisms has primarily been conducted in laboratory settings [92]. Since bacteriocins are mainly used for biopreserving food, further studies in food model systems are crucial to understanding resistance frequency and its impact on food microflora. The composition of food systems can affect bacteriocin activity and resistance development [82]. Exploring the genetic determinants of resistance mechanisms can help develop strategies to enhance bacterial susceptibility and design new bacteriocins that circumvent resistance [138]. Since resistance can spread to other bacteriocins, particularly within the same subclass, it is vital to avoid widespread use across multiple industries and limit usage to where they are most effective [139]. Like antibiotic resistance, bacteriocin resistance can be equally detrimental, so careful consideration is necessary when using bacteriocins in daily applications.

## Figures and Tables

**Table 1:** Classification of bacteriocins.

Class	Features	Examples, Producers	Mechanisms of Action	Receptors	References
I	Lantibiotics (<5 kDa) peptides containing lanthionine and methyllanthionine	Nisin (*Lactococcus lactis*)	Membrane permeabilization by pore formation and disrupting of cell wall synthesis	Lipid II	[74]
IIa	Small (<10 kDa), heat-stable peptides with broad-spectrum activity against *Listeria* spp.	Pediocin PA-1 (*Pediococcus acidilactici*), sakacin A (*Latilactobacillus sakei*)	Membrane permeabilization by pore formation	Mannose permease (Man-PTS)	[73]
IIb	Two-component bacteriocin: two different peptides required to form an active poration complex	Plantaricin JK (*Lactiplantibacillus plantarum*)	Membrane permeabilization by pore formation	UppP (undecaprenyl pyrophosphate phosphatase)	[75]
IIc	Circular bacteriocins	Enterocin AS-48 (*Enterococcus faecalis*)	Membrane permeabilization by pore formation	ABC transporter	[76]
III	Large protein (>30 kDa), heat-sensitive; limited application due to instability	Helveticin J (*Lactobacillus helveticus*)	Cell wall lysis through hydrolysis	Glycopeptides in cell wall	[77]

**Table 2:** The main mechanisms and genetic determinants associated with *L. monocytogenes* resistance to LAB bacteriocins.

Type of Modification	Mechanisms	Genetic Determinants	References
Changes in receptors	Shielding lipid II	*pbp2229*	[98]
	Mutational change in receptor structure	*mptACD* operon	[61]
Cell wall modification	Increased positive charges in cell wall-D-alanylation of teichoic acid or lipoteichoic acid	*dlt* operon	[103]
		*lmo1967* locus (homologue to *telA*)	[100]
Cell membrane modification	Changes in membrane fatty acid composition	*mprF*	[103,104]
	Increase in L-lysine content of membrane phospholipids		
	Divalent and trivalent cations stabilize the cytoplasmic membrane	N/A	[105]
	Change in membrane fluidity	*lmo2552* and *lmo1539*	[106]
Regulatory networks	Regulation of *mptACD* operon	*σB*	[106]
		*rpoN*	[107]
		*manR*	[108]
Two-component systems	Adaptation to the presence of bacteriocins	VirRS	[109]
		AnrAB	[100]
		LisRK	[110]
		LiaFSK	[110]

N/A—not applicable.

## Data Availability

No new data were created or analyzed in this study.
